# Comprehensive analysis of a NAD**+** metabolism-derived gene signature to predict the prognosis and immune landscape in endometrial cancer

**DOI:** 10.17305/bb.2023.9489

**Published:** 2024-04-01

**Authors:** Dan Hu, JunHong Du, YueMei Cheng, YiJuan Xing, RuiFen He, XiaoLei Liang, HongLi Li, YongXiu Yang

**Affiliations:** 1 The First School of Clinical Medicine, Lanzhou University, Lanzhou, China; 2 Department of Gynecology, The First Hospital of Lanzhou University, Lanzhou, China; 3 Key Laboratory for Gynecologic Oncology Gansu Province, Lanzhou, China

**Keywords:** Nicotinamide adenine dinucleotide (NAD+), endometrial cancer (EC), prognostic signature, biomarker, immunotherapy

## Abstract

As a crucial regulator influencing tumor progression, nicotinamide adenine dinucleotide (NAD**+**) is widely acknowledged. However, its role in endometrial cancer (EC) is not completely understood. In this study, we aimed to develop an NAD**+** metabolic-related genes (NMRGs) risk signature that could reflect the prognosis of EC patients and their responsiveness to immunotherapy and chemotherapy. Data from The Cancer Genome Atlas (TCGA) databases and the Molecular Signatures Database (MSigDB) confirmed two distinct NMRG subtypes in EC patients using consensus clustering, and a risk score was constructed utilizing an NAD**+**-related prognostic signature depending on the least absolute shrinkage and selection operator (LASSO) Cox regression analysis. Receiver operating characteristic (ROC) curves were employed to assess the model’s precision. Additionally, we used Gene Set Enrichment Analysis (GSEA) to predict the biological signaling pathways that might be involved. We also explored the role of the risk score in immune cell infiltration, tumor mutation burden (TMB), immunotherapy, and chemotherapy. Our study established a prognostic risk signature based on six NMRGs, and we observed that the high-risk group was associated with a poorer prognosis. Furthermore, we identified a strong correlation between the high-risk group and several pathways, including DNA replication, cell cycle, and mismatch repair. Lastly, our findings highlighted the influence of NMRGs on the regulation of immune infiltration in EC. Therefore, this signature holds potential value in predicting the prognosis of EC patients and guiding their management, including decisions regarding immunotherapy and chemotherapy, ultimately improving the accuracy of EC patient care.

## Introduction

Endometrial cancer (EC) is the second most common gynecological cancer globally. In 2020, there were roughly 400,000 new cases and over 80,000 recorded fatalities worldwide, as reported by the American Cancer Society’s latest available statistics [1]. Traditional pathology divided EC into type 1 and type 2 [2]. Type 1 accounted for about 70%–80% of ECs, consisting of moderately or well-differentiated endometrioid tumors with positive hormone receptors and had a favorable prognosis. Type 2 tumors, which made up 20%–30% ECs, had a poor prognosis, a poorly differentiated histology, and lacked hormone receptors. The evaluation of histological characteristics like gradation, histotype, depth of myometrial invasion, and involvement of neighboring tissues like the cervix and annexes remained a limitation of EC risk categorization for decades. Fortunately, in 2013, The Cancer Genome Atlas (TCGA) Research Network overcame the limitations of EC classification by integrating molecular characterization and proposed for the first time a molecular typing of EC, including four subtypes: POLE (ultramutated), MSI (hypermutated), copy-number low (endometrioid), and copy-number high (serous-like) [3]. Each group has a unique relationship to progression-free survival and recurrence risk. Even though the majority of EC patients can be identified and treated at early stages, 15% of EC patients are identified at a locally advanced or occult metastatic stage and experience tumor recurrence due to a limited response to surgery and radiation therapy [4]. Therefore, it is crucial to discover predictive indicators for EC and to investigate medications that are more efficient for individuals with advanced forms of the disease.

Metabolic reprogramming, an important hallmark of malignant tumors, gives growth and proliferative latent energy to cancer cells in the nutrient-impoverished tumor microenvironment (TME) through modifying metabolism [5]. Nicotinamide adenine dinucleotide (NAD+) is an important coenzyme in oxidative reactions and is also essential for cellular homeostasis, cell proliferation, cell death, genomic stability, and immunological responses [6–8]. During the process of glycolysis, NAD+ can be regenerated by lactate dehydrogenase (LDH) reaction in the cytosol, which promotes the growth of tumor cells. Cancer cells have higher proportions of NADP+/NADPH and NAD+/NAD, indicating the critical involvement of NAD+ in this metabolic conversion when compared to non-cancerous cells [9–11]. In addition, NAD+ also functions as a key cofactor for non-oxidative NAD+-dependent enzymes, such as poly (ADP-ribose) polymerases (PARPs), cADPRSs, and sirtuin in numerous signaling pathways, including DNA repair, post-translational changes, inflammatory responses, senescence, and apoptosis [12–14]. Therefore, improving the quality of patient survival is feasible by exploring the function of NAD+ metabolism-related genes (NMRGs) in EC and making it a new target instead of ineffective traditional anti-cancer treatments.

There is growing evidence that NAD+ metabolism-derived gene signatures exhibit strong predictive potential across a variety of tumor types, including ovarian cancer and cervical cancer [15, 16]. However, no research on the NMRGs has been published concerning the prognostic evaluation of EC. In this study, we thoroughly analyzed NMRGs to investigate the impact of NAD+ metabolism on the survival and immune landscape of EC patients. Moreover, we developed a risk score model based on NMRGs to assess their predictive significance in EC. Our research may offer a fresh perspective on the molecular underpinnings behind EC, bring new insight to the targeting therapy approach for EC, and promote the individual-based treatment of EC patients.

## Materials and methods

### Data collection and preparation

We obtained mRNA expression profiling (FPKM format) data and corresponding clinicopathological information, which included 552 EC samples and 35 normal samples from TCGA (http://cancergenome.nih.gov) database. Patients without complete clinical data were excluded from the analysis. In total, 540 eligible EC samples were taken from 552 patients for the follow-up study. Table S1 lists the clinical characteristics of the 540 EC patients.

### Selection of NAD**+ **metabolic-related genes (NMRGs) and functional enrichment analysis

We obtained gene sets associated with NAD+ from the Molecular Signature Database (MSigDB) [17], which includes the Reactome database (R-HSA-196807) and the Kyoto Encyclopedia of Genes and Genomes (KEGG) pathway database (Pathway: hsa00760). Forty-two NMRGs were collected for differential analysis with log fold change > 1 and false discovery rate (FDR) < 0.01 as a filter using the R limma package. The R “ClusterProfiler” package was used to conduct KEGG and Gene Ontology (GO) studies.

### Consensus clustering analysis

Based on the expression of NMRGs, an unsupervised consensus clustering analysis was applied to classify EC patients into the optimal number of clusters using the “ConsensusClusterPlus” package of R. Next, we performed Kaplan–Meier and log-rank tests to acquire the overall survival (OS) statistics.

### Establishment and validation of the prognostic signature

The EC samples were randomly split into a training cohort and a validation cohort for bioinformatics analysis with a proportion of 6:4. Univariate analysis was used to identify potential NMRGs associated with the prognosis of EC in the training cohort. In our study, we used the OS as the clinical parameter of prognosis. After that, by least absolute shrinkage and selection operator (LASSO) regression analysis with 10-fold cross-validated results, we eliminated several strongly linked genes to avoid the overfitting impact. Finally, we identified six genes (*SLC22A13*, *CYP8B1*, *NMRK1*, *NAXE*, *NT5E*, and *NT5M*) strongly associated with prognosis in EC patients screened by LASSO regression and established the prognostic risk signature using regression coefficients. The risk score was assessed using the following formula:


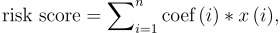


where *n*, *x*, and coef represent the number of genes, expression value, and coefficient, respectively.

Based on the median risk score, the subgroups of high-risk and low-risk EC patients were determined. Log-rank tests and Kaplan–Meier survival analyses were used to verify the correlation between OS and risk score. The receiver operating characteristic (ROC) curve and the area under the curve (AUC) were used to evaluate the prognostic potential of the risk score using the “timeROC” R package. The survival state diagram and risk curve were created using the “ggrisk” R tool. Additionally, the validation cohort and the entire cohort both verified the ROC curve, Kaplan–Meier survival analysis, and survival state diagram. Regression models using univariate and multivariate data were applied to identify independent prognostic elements in EC.

### Establishment and validation of a nomogram

The nomogram was created using the “rms” R tool to incorporate several prediction indicators based on multivariate Cox regression studies. After that, we used calibration plots to assess the nomogram’s dependability.

### Gene Set Enrichment Analysis (GSEA)

The characteristic gene sets may have been differently enriched in each group, according to GSEA [17]. For the full cohort, we compared the biological processes (BPs) that were markedly different between different clusters and risk groups using GSEA software (version 4.1.0). FDR < 0.25 and *P* < 0.05 were intended to indicate a meaningful difference.

### Tumor mutation burden (TMB) analysis

The TCGA GDC Data Portal provided somatic mutation information for the EC samples in “maf ” format. We utilized the “Maftools” package to generate waterfall plots, facilitating the visualization and summarization of the mutated genes. The formula below was used to evaluate the TMB score:


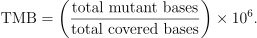


### Immunogenomic landscape analysis

To compare the variations in the TME between various clusters and risk groups, in light of TCGA RNA-sequencing data, we evaluated 22 immune cells’ infiltration by the CIBERSORT algorithm. The ESTIMATE method was used to compute the immunoscore by the R “estimate” package. The two-sample Wilcoxon test was used to examine immune infiltration and function among the various groups. Also, we looked at how often expressed immune checkpoint inhibitor (ICI) genes were in the high- and low-risk categories. The threshold for statistical significance was *P* < 0.05.

### Immunotherapy response prediction

The Cancer Immunome Atlas (TCIA, https://tcia.at/) provided the immunophenoscore (IPS) for the EC samples, which can forecast the effectiveness of immunotherapies, such as programmed death-ligand 1 (PD-L1), PD-L2, programmed cell death protein 1 (PD-1), and cytotoxic T-lymphocyte associated protein 4 (CTLA4) blockers. The IPS score was standardized to have a range from 0 to 10, with a higher IPS score denoting greater immunological reactivity. The potential outcomes of immune checkpoint blockade reactions in EC were predicted using the Tumor Immune Dysfunction and Exclusion (TIDE) technique. A lower TIDE score denotes a greater immunotherapy response.

### The response to chemotherapy and small-molecule drugs

Genomics of Drug Sensitivity in Cancer (GDSC, www.cancerrxgene.org) is a public database that evaluates the therapeutic potential of chemotherapy and small-molecule medicines. We utilized the “pRRophetic” and “ggplot2” R packages to compare the half-maximal inhibitory concentration (IC50) of various chemotherapeutic and small-molecule medications for EC between the high- and low-risk groups to ascertain the drug sensitivity.

### Ethical statement

The data sourced from the public database are open access, so there was no requirement for the approval of a clinical ethics committee. The study adhered to the corresponding rules of the public database. 

### Statistical analysis

All data visualization and statistical analysis were conducted using R 4.2.0 (http://www.r-project.org). Wilcoxon’s test was used to identify the differentially expressed genes (DEGs) between EC cases and controls. The variations in survival across the various subtypes of EC patients were assessed using log-rank tests and Kaplan–Meier analyses. The cutoff point for statistical significance was < 0.05.

## Results

### Identification and functional enrichment analysis of NMRGs

**Figure 1.**
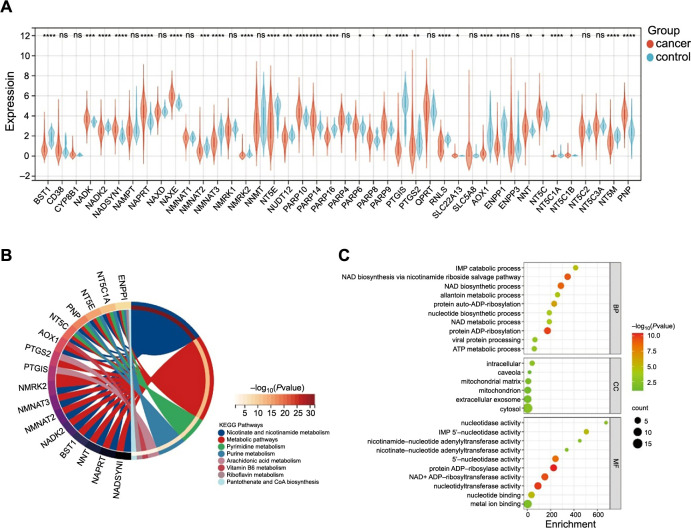
**Identification of DEGs related to NAD+ metabolism of TCGA datasets between EC and normal tissues and GO and KEGG pathway enrichment analysis of DEGs.** *P < 0.05, **P < 0.01, ***P < 0.001, and ****P < 0.0001; (A) Differential expression of NAD+ metabolism-related genes in EC (*n* ═ 540) and controls (*n* ═ 35); (B) KEGG pathway analysis of DEGs; (C) GO functional enrichment analysis of DEGs includes three domains: molecular function, biological process, and cell composition. DEG: Differentially expressed gene; NAD+: Nicotinamide adenine dinucleotide; TCGA: The Cancer Genome Atlas; EC: Endometrial cancer; GO: Gene Ontology; KEGG: Kyoto Encyclopedia of Genes and Genomes; BP: Biological process; CC: Cell composition; MF: Molecular function.

The analysis process for the study is shown in Figure S1. We obtained 42 NMRGs (Table S2) for an examination of the differential expression between EC and normal tissues. Finally, 29 DEGs with log fold change > 1 and FDR < 0.01 were retained for further analysis, including 14 upregulated and 15 downregulated genes (Figure 1A). Additionally, we validated the biological role and signaling pathways of DEGs using GO and KEGG enrichment analysis, and discovered that in the KEGG pathways, the DEGs were mainly involved in nicotinate and nicotinamide metabolism, nucleotide metabolism, and pyrimidine metabolism (Figure 1B). In the biological process (BP), the DEGs were primarily involved in NAD biosynthesis via the nicotinamide riboside salvage pathway, NAD biosynthetic process, and protein ADP-ribosylation. In the molecular function (MF), the DEGs were primarily involved in 5’-nucleotidase activity, protein ADP-ribosylase activity, nucleotidyltransferase activity, and NAD+ ADP-ribosyltransferase activity. In the cell components (CCs), the DEGs were primarily involved in the cytosol, mitochondrial matrix, and intracellular composition (Figure 1C).

### Consensus clustering of NMRGs distinguished two clusters of endometrial cancer (EC) with different prognoses

**Figure 2.**
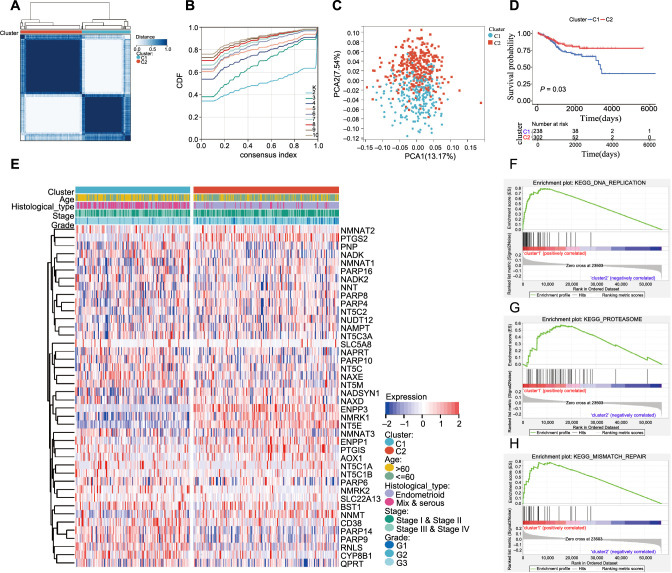
**Differential clinicopathological features and survival of EC patients in two clusters in TCGA cohort.** (A) TCGA EC group was assigned into two clusters when k ═ 2; (B) Consensus clustering cumulative distribution function (CDF) for k ═ 2–10; (C) PCA analysis confirmed the classification; (D) Kaplan–Meier OS curve for EC patients in the clusters (C1 ═ 238 and C2 ═ 302); (E) The distribution of clinicopathological variables between different clusters (C1 ═ 238, C2 ═ 302); and (F–H) GSEA for cluster 1. DNA replication (F), proteasome (G), and mismatch repair (H) were significantly enriched in cluster 1. EC: Endometrial cancer; TCGA: The Cancer Genome Atlas; PCA: Principal component analysis; OS: Overall survival; GSEA: Gene Set Enrichment Analysis.

Based on the similarity of NMRG expression, we classified EC patients using consensus clustering. It was determined that k ═ 2 was a suitable criterion for separating EC patients into two subgroups (Figure 2A and 2B). According to the principal component analysis (PCA), the NMRG clusters were split into two distinct clusters (Figure 2C). According to the survival analysis results (Figure 2D), patients in cluster 2 had significantly better outcome (*P* ═ 0.03). Then, we assessed the association between cluster and clinicopathological traits and visualized it as a heatmap. We found significant differences in histological type and pathological grade in the two clusters (Figure 2E). Patients in cluster 2 were characterized by younger age, lower cancer stage and grade, and a predominant histological type of endometrioid (Figure S3A–S3D). Then, we conducted GSEA enrichment analysis, and the results depicted that pathway like “DNA replication,” “proteasome,” and “mismatch repair” were primarily enriched in cluster 1 (Figure 2F–2H).

**Figure 3.**
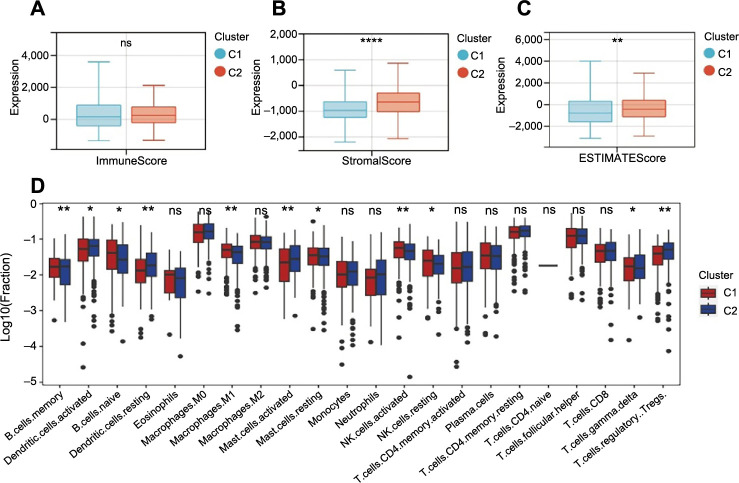
**The relationship between clusters assignment and (A) immune score, (B) stromal score, (C) ESTIMATE score, and (D) the landscape of immune cell infiltration in EC (C1 ═ 238 and C2 ═ 302).** *P < 0.05, **P < 0.01, and ****P < 0.0001. EC: Endometrial cancer.

### Consensus clustering for NMRGs associated with immunocyte infiltration

We computed the immune score, stromal score, and ESTIMATE score of the two clusters to analyze the association between the TME and NMRGs. In cluster 1, there was a downregulation of the stromal and ESTIMATE score compared to cluster 2, except for no difference in immune score (Figure 3A–3C). Then, we compared the immunological infiltration of 22 various immune cell subtypes between two clusters using CIBERSORT. The findings from 540 EC patients in the TCGA are summarized in Figure 3D. Cluster 1 had lower infiltration levels of naive B cells, regulatory T cells (Tregs), resting dendritic cells, and activated mast cells, whereas it had higher infiltration levels of memory B cells, M1 macrophages, resting NK cells, activated NK cells, activated dendritic cells, gamma delta T cells, and resting mast cells.

### Construction and validation of the NMRGs risk signature

**Figure 4.**
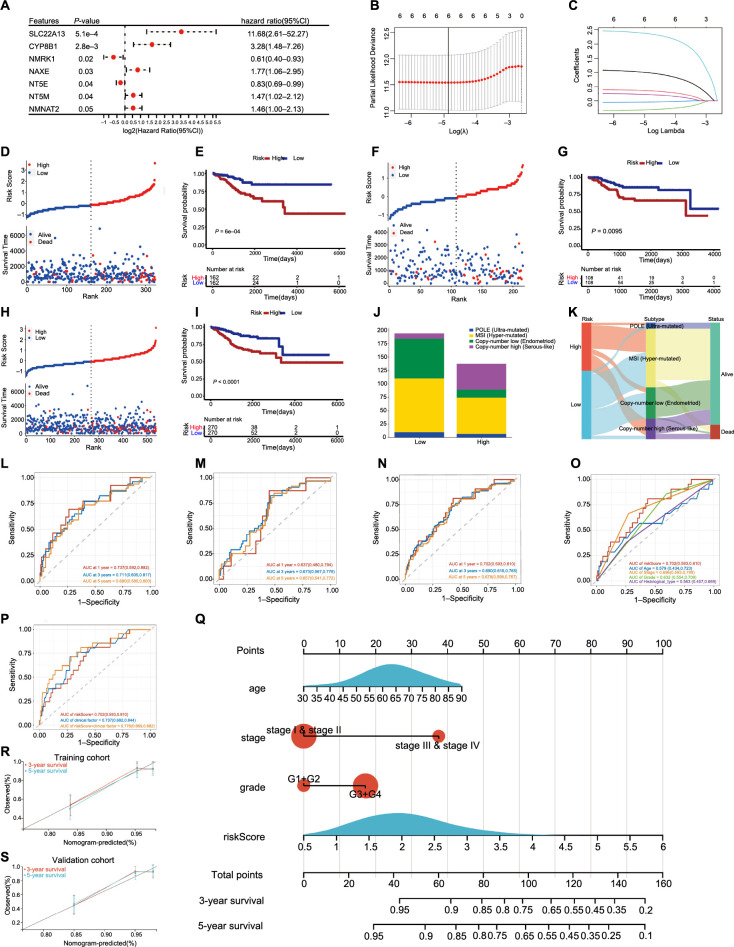
**Construction and verification of risk signatures associated with NMRGs in EC.** (A) Results of univariate Cox analysis for the prognostic genes; (B and C) LASSO regression model; (D) The distribution of risk score and OS status in the training cohort (High ═ 162, Low ═ 162); (E) Survival analysis in the training cohort (High ═ 162, Low ═ 162); (F) The distribution of risk score and OS status in the validation cohort (High ═ 108, Low ═ 108); (G) Survival analysis in the validation cohort (High ═ 108, Low ═ 108); (H) The distribution of risk score and OS status in the entire cohort (High ═ 270, Low ═ 270); (I) Survival analysis in the entire cohort (High ═ 270, Low ═ 270); (J and K) The vertical stack bar and Sankey diagram of molecular classification of EC in different risk groups (High ═ 137, Low ═ 194); (L) The time-dependent ROC curve in the training cohort; (M) The time-dependent ROC curve in the validation cohort; (N) The time-dependent ROC curve in the entire cohort; (O) The time-dependent ROC curves for risk score and clinical factors with 1-year OS in the entire cohort; (P) The time-dependent ROC curves for clinical factors (age and stage) and clinical factors + risk score with 1-year OS in the entire cohort; (Q) Nomogram to predict 3-year and 5-year OS; (R and S) Calibration plots of 3-year and 5-year OS for nomograms in the training cohort and validation cohort. NMRG: NAD+ metabolic-related gene; EC: Endometrial cancer; LASSO: Least absolute shrinkage and selection operator; OS: Overall survival; ROC: Receiver operating characteristic.

By integrating mRNA expression profiles with OS data, we screened out 540 OS-related prognostic EC samples. The EC samples mentioned above were split into training and validation cohorts in 6:4 ratio. After doing univariate regression analysis in terms of OS in the training cohort, we screened seven genes that might forecast the prognosis of EC and found that they all met the condition of being closely related to OS (*P* < 0.05) (Figure 4A). After that, LASSO regression analysis was used to prevent overfitting and create the EC prognostic model (Figure 4B and 4C). Finally, risk signatures for six genes were discovered (*SLC22A13, CYP8B1, NMRK1, NAXE, NT5E*, and *NT5M*). The following algorithm serves as the foundation for the risk score model: Risk Score ═ (2.435) * *SLC22A13* + (1.056) * *CYP8B1* + (−0.333) * *NMRK1* + (0.388) * *NAXE* + (−0.049) * *NT5E* + (0.250) * *NT5M*.

Based on the median risk score, the training cohort’s samples were split into two groups. The survival status of EC patients revealed that the proportion of alive statuses in the low-risk group was much higher than that in the high-risk group (Figure 4D). The Kaplan–Meier analysis revealed that the high-risk group had a worse prognosis (Figure 4E), which was further supported by equivalent results in the validation cohort and the entire cohort (Figure 4F–4I). On this basis, we synthesized the molecular classification of EC and further evaluated the prognosis of different risk groups. The results showed that the percentage of MSI and copy-number low in the low-risk group was higher than the high-risk group, and the percentage of copy-number high was significantly lower than in the high-risk group (Figure 4J and 4K). This might explain why the prognosis of patients in the low-risk group is better at a deeper level from the molecular aspect. ROC curve analysis was used to assess the signature’s predictive power, with AUC values 0.737, 0.711, and 0.690 at one, three, and five years in the training cohort, respectively (Figure 4L). Figure 4M shows AUC values 0.637, 0.673, and 0.657 at one, three, and five years in the validation cohort, respectively. AUC values 0.702, 0.690, and 0.678 at one, three, and five years in the entire cohort, respectively, are shown in Figure 4N. The research discussed above further demonstrated that the sensitivity and reliability of the signature for predicting the prognosis of EC patients ranged from low to moderate. We also used ROC curve analysis in the entire cohort to verify the predictive power of risk score and clinicopathological factors alone and in combination. In comparison to other clinicopathological variables, the risk score’s AUC value was much higher (Figure 4O), and the AUC for risk score + clinical factor is much more favorable for postoperative EC patients (Figure 4P). Besides that, age, stage, and risk score were further revealed to be independent prognostic factors in patients with EC by the findings of univariate and multivariate Cox analyses of training, validation, and the entire cohort, although there was no difference in HR among them (Figure S4A and S4B and Table S3).

In order to quantify the prognostic risk assessment of EC patients, we developed a predictive nomogram to estimate more accurately the OS time by combining clinicopathological traits associated with prognosis (Figure 4Q). According to the sum of all parameters, we projected the three-year and five-year survival rates of EC patients. The prediction lines of the nomogram for three- and five-year survival probability in the calibration study were very close to the ideal performance, indicating a high degree of accuracy of the nomogram for properly forecasting the three-year and five-year survival rates of diagnosed patients (Figure 4R and 4S).

### Functional analysis of the risk score

**Figure 5.**
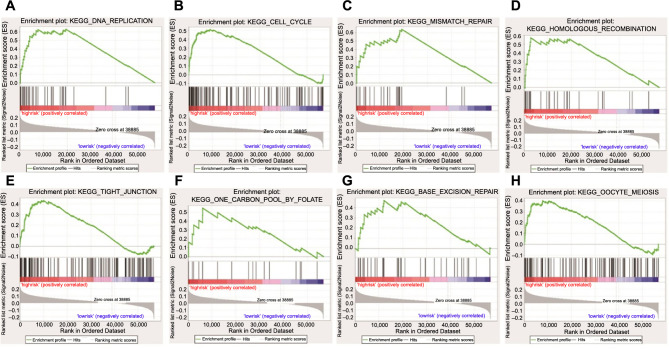
**The enriched biological pathways from GSEA.** (A) DNA replication; (B) Cell cycle; (C) Mismatch repair; (D) Homologous recombination; (E) Tight junction; (F) One carbon pool by folate; (G) Base excision repair; (H) Oocyte meiosis. GSEA: Gene Set Enrichment Analysis.

GSEA was conducted between tissues in various risk groups. The FDR *q*-value <0.25, normalized *P* value < 0.05, and normalized enrichment score were used to select the enriched biological pathways. The high-risk group was associated with several BPs, including DNA replication, mismatch repair, cell cycle, homologous recombination, oocyte meiosis, tight junction, one carbon pool by folate, and base excision repair (Figure 5A–5H).

### Association of TMB with NAD+ metabolism-related risk score

**Figure 6.**
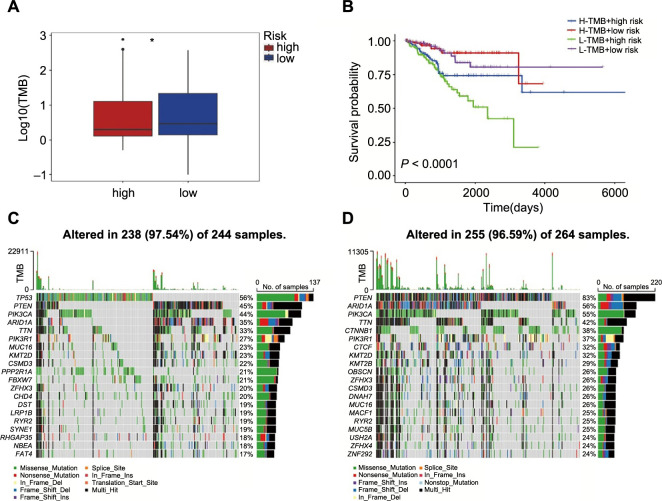
**Comparison of somatic mutations between different risk groups.** *P < 0.05; (A) The association of TMB with risk score (High ═ 244, Low ═ 264) by applying the Mann–Whitney *U* test; (B) The Kaplan–Meier curve survival analysis for EC patients stratified by both TMB groups and risk groups; (C and D) Waterfall plot displaying gene mutations in the high- (C) and low-risk (D) groups. TMB: Tumor mutation burden; EC: Endometrial cancer.

TMB has recently been shown as a biomarker that predicts response to immunotherapy in several malignancies [18–20]. TMB levels reflect the repair damage to DNA within tumor cells and are closely correlated with the ability to create tumor neoantigens. Figure 6A shows that persons at low risk had higher TMB levels. Additionally, patients may be divided into four groups using the TMB status and risk score, and each group had a distinct prognostic outcome (Figure 6B). The top 20 genes’ mutation differences among various risk categories in EC were displayed as a waterfall plot, where various mutation types were represented by various colors on the bottom (Figure 6C and 6D). Except for *PIK3CA*, *PIK3R1, KMT2D*, and *RYR2*, the ranking and mutation rates of the other 16 genes were different in the high- and low-risk groups. Meanwhile, the low-risk group of patients had a higher mutation frequency, which was in line with the TMB score. These mutations in all risk groups were further classified and summarized according to the classification category, with missense mutations accounting for the largest proportion of all risk groups (Figure S5A). SNP occurred more frequently than the other three variation types (Figure S5B). The most prevalent single nucleotide variant class was C > T (Figure S5C).

### The link between immune cell infiltration and NAD+ metabolism-related risk score

**Figure 7.**
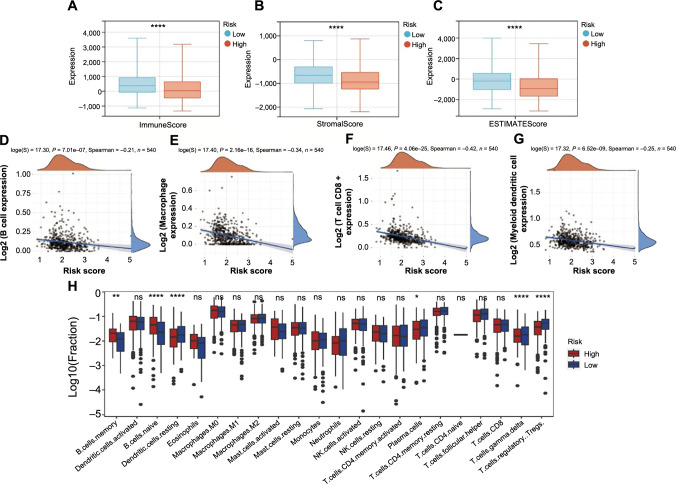
**The relationship between risk score and distinct immune cell infiltration.** *P < 0.05, **P < 0.01, and ****P < 0.0001; (A–C) Comparison of TME scores between low- (*n* ═ 270) and high-risk (*n* ═ 270) groups; (D–G) The Spearman correlation between risk score and the fraction of peripheral immune cells is shown; Scatterplots depicts that risk score is negatively correlated with B cells (D), macrophages (E), CD8+ T cells (F), and myeloid dendritic cells (G); (H) Comparison of the infiltration of 22 immune cells between low- (*n* ═ 270) and high-risk (*n* ═ 270) groups. TME: Tumor microenvironment.

Recent research has shown that the immunology of tumors is related to the regulation of metabolic pathways [21]. Given the significant biological roles of NAD+ in antitumor immunological responses, we assessed immunoscore and immune infiltration levels in the full TCGA cohort to further understand the effect of risk score on the immunological microenvironment of EC. The results demonstrated that immune, stromal, and ESTIMATE scores were downregulated in the high-risk group (Figure 7A–7C). The immune correlation analysis revealed a negative association between the risk score and B cells (Figure 7D), macrophages (Figure 7E), CD8+ T cells (Figure 7F), and myeloid dendritic cells (Figure 7G). Additionally, the high-risk group contained fewer immune infiltrating cells, such as resting dendritic cells, T cells CD4 memory activated, gamma delta T cells, and Tregs, according to the results of the immune cell infiltration analyses (Figure 7H). These findings demonstrated a connection between the risk score related to the NAD+ metabolism and distinct immune cell infiltration.

### Connection of immune checkpoint genes and mRNAsi with NAD+ metabolism-related risk score

**Figure 8.**
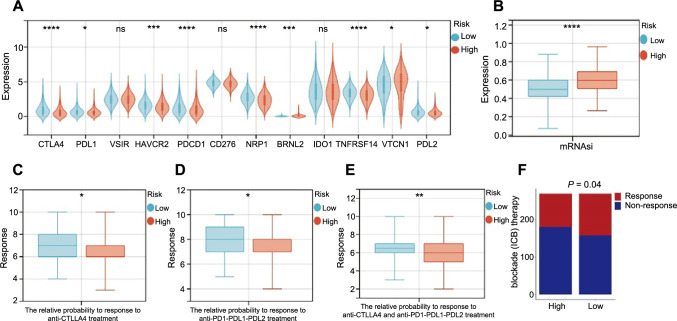
**The relationship between risk score and immune checkpoint molecules.** *P < 0.05, **P < 0.01, ***P < 0.001, and ****P < 0.0001; (A) The association between risk score and ICI genes (High ═ 270, Low ═ 270); (B) The association between risk score and mRNAsi (High ═ 270, Low ═ 270); (C–E) The prediction of immunotherapy response (High ═ 270, Low ═ 270); (F) The relative probabilities to respond to immunotherapy (High ═ 270, Low ═ 270). ICI: Immune checkpoint inhibitor.

ICI has emerged as a viable treatment for several cancers. The expression of common ICI genes was first compared between the two risk groups and found that most immune checkpoint genes (*CTLA4, PDL1, PDL2,* hepatitis A virus cellular receptor 2 [*HAVCR2*], programmed cell death 1 [*PDCD1*], neuropilin 1 [*NRP1*], and TNF receptor superfamily member 14 [*TNFRSF14*]) were substantially elevated in low-risk patients (Figure 8A). The NMRG risk score’s capacity to forecast the ICI response was further tested by conducting an IPS analysis to ascertain the immunotherapeutic sensitivity of EC patients. As shown in Figure 8C–8E, the possibilities of response to anti*-PD-1/PD-L1/PD-L2* and anti-*CTLA4* treatment were higher in low-risk patients. The expression levels of mRNAsi in patients with low-risk scores were also lower (Figure 8B). This suggests stronger immunogenicity in low-risk patients and more responsiveness to immune checkpoint blockade therapy. The TIDE algorithm confirmed our findings that immunotherapy was more effective for people in the low-risk group (Figure 8F).

### Association of chemotherapy and small-molecule drugs with NAD+ metabolism-related risk score

**Figure 9.**
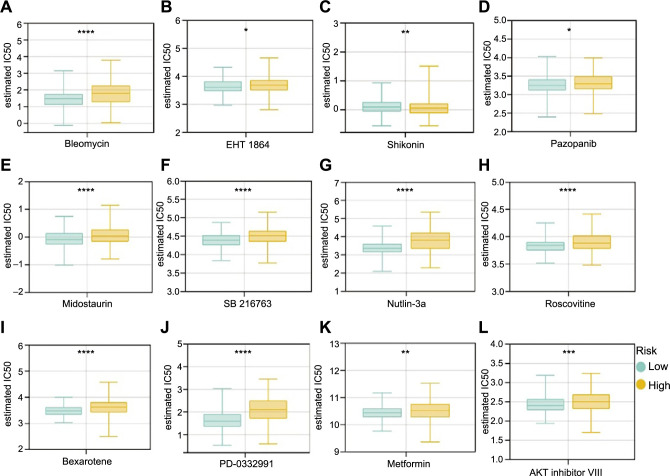
**Analysis of drug sensitivity in high- (*n* ═ 270) and low-risk (*n* ═ 270) groups.** *P < 0.05, **P < 0.01, ***P < 0.001, and ****P < 0.0001; (A) Bleomycin; (B) EHT-1864; (C) Shikonin; (D) Pazopanib; (E) Midostaurin; (F) SB 216763; (G) Nutlin.3a; (H) Roscovitine; (I) Bexarotene; (J) PD-0332991; (K) Metformin; and (L) AKT inhibitor VIII.

Since chemotherapy and small-molecule medications are frequently used to treat EC, the GDSC database was utilized to assess the strength of the effects of these treatments. We evaluated the IC50 of popular chemotherapeutic and small-molecule medications between high-risk and low-risk patients and discovered that, with the exception of Shikonin, most medications had lower IC50 scores in low-risk patients, indicating low-risk patients were more sensitive to these medications (Roscovitine, Bleomycin, Bexarotene, Pazopanib, Metformin, Midostaurin, PD-0332991, EHT-1864, SB 216763, AKT inhibitor VIII, and Nutlin.3a) (Figure 9A–9L). The molecular targets and targeting pathways of these drugs are shown in Table S4.

**Figure 10.**
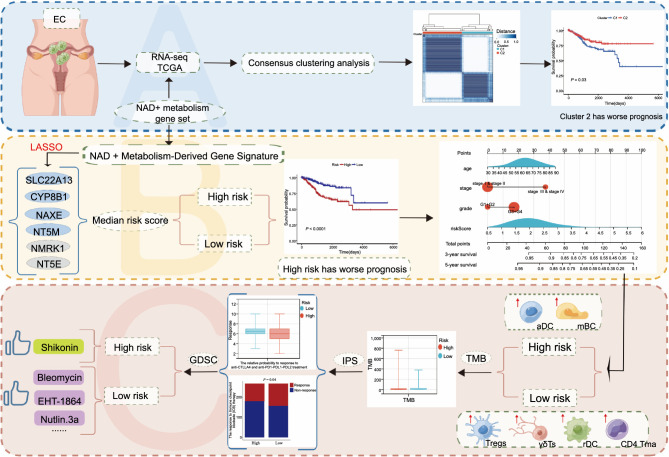
**The concluding figure highlighting the importance of the NAD+ metabolic-derived gene signature for the prognosis, immune microenvironment, and treatment in EC patients.** NAD+: Nicotinamide adenine dinucleotide; EC: Endometrial cancer.

## Discussion

While in the past ten years, a significant increase in the use of histo-pathological tumor features for therapeutic decision making has been seen, genetic subtype-based diagnostic and prognostic techniques are emerging quickly and have made advancements in the choice of targeted adjuvant therapy [22]. NAD+ is a key coenzyme in oxidative reactions that directly or indirectly participates in energy metabolism, cellular senescence, DNA repair, immune cell function, and chromatin remodeling [23–26]. Numerous studies have found links between the dysregulation of NAD+ levels and metabolic disorders and aging-related diseases, such as cancer, neurodegeneration, and defective immune responses. As a result, there is a resurgence of curiosity about how the NAD+ metabolism affects the origin of diseases, including cancer. Moreover, preclinical research and clinical trials in cancers including gastric cancer, glioblastoma, melanoma, and chondrosarcoma all showed the effectiveness of NAD+ biosynthesis-targeted inhibitors as anticancer treatments [27–30]. In this study, we built a NAD+ metabolism gene signature to provide suggestions for both risk-based prognosis and targeted adjuvant therapy of EC patients.

Two cluster subtypes were characterized by the expression similarity of NMRGs, with cluster 2 having a higher chance of survival. Meanwhile, the results of GSEA enrichment analysis suggested that pathways like “DNA replication,” “proteasome,” and “mismatch repair” were primarily enriched in cluster 1. DNA replication is a crucial process for the accurate replication and transmission of genetic information, and its errors result in replication stress which is a prerequisite for the molecular and clinical development of malignancies [31]. Proteasomes, which are characterized by trypsin-, chymotrypsin-, and caspase-like activities, are crucial for intracellular protein breakdown and, hence, play a significant role in the pathophysiology of many disorders, including their function in the emergence and growth of malignant tumors. Spirina et al. [32] found that the progression of EC is associated with an increase in total proteasome activity. In addition to this, a recent study discovered that the combination of the proteasome inhibitor ixazomib and the HDAC inhibitor romidepsin significantly increased cell death in patient-derived organoid models and cell lines in various gynecological malignancies, including EC. This study provided a new therapeutic strategy to improve the prognosis of patients with gynecologic cancers [33]. Defective DNA mismatch repair is one of the most prevalent and best characterized genetic abnormalities detected in EC, occurring in approximately 20%–25% of all cases [34]. Arabi and associates provided the findings of a study in which they evaluated the level of DNA mismatch repair in high-grade ECs. Loss of DNA mismatch repair may be linked to unfavorable outcomes, according to their research of 91 instances [35].

The tumor immune microenvironment (TIME) has been referred to as the “fertile soil” for the malignant transformation of tumors. The infiltrating stromal and immune cells, as the main elements of TIME, are crucial in the biology of cancer [36]. TIME also plays a key role in controlling the NAD+ metabolism and homeostasis. Many cell types, such as B cells, T cells, and macrophages, are involved in maintaining the NAD+ metabolism [37–39]. The lack of stromal and immune cells in tumor tissues was explained by a significantly lower stromal score and ESTIMATE score derived from cluster 1, which pointed to a more complicated TME and a subpar clinical outcome. A recent study showed that TME composition affects the clinical outcome of EC patients, with a worse prognosis for patients with low immune and stromal scores [40], which is consistent with our results. Among the 22 common immune infiltrating cells, Treg is a key factor in the induction of tumor immune tolerance in various cancers. A recent study showed that high infiltration of Treg improves the prognosis of EC patients, and Liu et al. also found that high levels of Treg and naive B cell infiltration were associated with longer OS and recurrence-free survival (RFS) in EC, which may explain the worse prognostic outcome in cluster 1 with low infiltration of Treg and naive B cell in our study [41, 42].

Six NMRGs (*SLC22A13, CYP8B1, NMRK1, NAXE, NT5E*, and *NT5M*) were chosen as the predictor variables in the prognostic gene signature for a calculation of risk score for the EC patients from the TCGA. The ROC curve analysis further demonstrated that the risk score had a higher predictive value than any other clinical factors currently in use and the united AUC for risk score and that the clinical factor was much more favorable for postoperative EC patients. Therefore, we combined a number of prognostic variables (age, stage, grade, risk score, and survival rate) to build a nomogram that accurately predicts the patient’s three five-year survival rate, which may aid in the planning of short-term follow-up of customized treatment.

Then, we used GSEA to explore the biological functions of six genes in EC and discovered that the high-risk EC group involves DNA replication, mismatch repair, cell cycle, homologous recombination, tight junction, one carbon pool by folate, base excision repair, and oocyte meiosis. Cell cycle dysregulation is among the genetic alterations underlying the development and progression of EC [43]. Liu et al. [44] developed a novel cell cycle-related prognostic signature that can accurately predict the prognosis of EC patients. In parallel, the potential of cell cycle checkpoint inhibitors as therapeutic targets has been demonstrated in a variety of cancers, including EC, breast cancer, and ovarian cancer [45–47]. The homologous recombination pathway is a repair pathway with high fidelity, and its functional defects increase the likelihood of normal cell carcinogenesis. A recent study conducted a large-scale assessment of the prevalence of homologous recombinant DNA damage repair (HR-DDR) genes by examining the molecular profiles of 52,426 tumor tissues. The results showed that *HR-DDR* is most frequently mutated in EC, providing an avenue to explore the role of HR-DDR deficiency targeted therapy in EC [48]. In addition, the tight junction pathway, base excision repair pathway, and oocyte meiosis pathway have been shown to be involved in the occurrence and development of EC, which is consistent with our findings [49–51].

TMB is recognized as a predictive biomarker of immune response and tumor biological behavior, and the accumulation of somatic mutations, one of the primary drivers of carcinogenesis, also promotes the production of neoantigens. According to our study, patients in the low-risk group had higher TMB levels than those in the high-risk group and had a poorer prognosis for patients with low TMB under the influence of risk score. Our risk signature revealed that somatic gene changes, particularly the *PTEN* mutant, were highly active whether a risk score was high or low. *PTEN*, a common tumor suppressor gene, whose deletion often occurs in concert with *CTNNB1* missense mutations and *PIK3CA* activation to promote myofibrillar infiltration, resulting in the formation of EC [52]. Meanwhile, the existence of *PTEN* mutation is highly related to positive prognosis and has an impact on EC patients’ survival [53, 54]. Our findings also revealed a significant mutation in the tumor suppressor gene *ARID1a*. There are reports that when *ARID1a* was deleted, *PTEN* boosted the cellular proliferation in the transition of precancerous lesions to EC [55], which may explain why the low-risk group has a favorable prognosis despite the highest frequency of *PTEN* and *ARID1a* mutations.

Growing research suggested that the abnormal NAD+ metabolism-derived gene expression level can cause immune system dysfunction [16, 56]. According to the result of the TME study in our article, patients with high risk have a significant decrease in ESTIMATE scores and have less enrichment of immune-infiltrating cells in their TME. Immune escape was more likely to occur in metastatic lesions with the fewest immunological cells present since this represented the worst immune microenvironment. These results indicated that the NAD+ metabolism was closely correlated with the immunogenomic landscape of EC and low levels of immune cell infiltration may cause poor prognosis in high-risk patients.

We also investigated if a gene-based risk signature for the NAD+ metabolism could convey important information regarding how immunotherapy and chemotherapy are reflected. The available data demonstrated that several particular metabolic pathways have a role in the response to immunotherapy [57–59]. The findings of this study showed that most immune checkpoint genes (*CTLA4, PDL1, PDL2, HAVCR2, PDCD1, NRP1*, and *TNFRSF14*) were highly expressed in low-risk patients. Additionally, we found that low-risk patients responded more sensitively to immunotherapy than high-risk patients, suggesting that immunotherapy may not be beneficial for high-risk patients. The results of the medication sensitivity study in the GDSC dataset showed that excepting Shikonin, patients with low risk responded better to chemotherapy or small-molecule medications like Roscovitine, Bleomycin, Bexarotene, Pazopanib, Metformin, Midostaurin, PD-0332991, EHT-1864, SB 216763, AKT inhibitor VIII, and Nutlin.3a. Given the current limited availability of these drugs in cancer research, future studies should emphasize investigating the potential benefits of these novel chemotherapy or small-molecule drugs in EC patients.

The study we conducted does have some limitations. First, the model’s performance in predicting the prognosis of certain EC patients may be compromised, as indicated by the suboptimal ROC outcomes in the validation cohort. Second, this study relies solely on bioinformatic analysis of the TCGA database, highlighting the necessity for experimental research and clinical investigations involving larger sample sizes to validate the predictive capacity of this signature.

## Conclusion

This study identified NAD metabolism-derived genes in EC patients. Furthermore, we created a NAD+ metabolism-derived gene model that can predict the outcomes of EC patients with relative accuracy based on six chosen NMRGs (Figure 10). This signature may contribute to the exploration of more effective immunotherapy strategies and the identification of novel molecular targets in EC.

## Supplemental data

Supplementary data are available at the following link: https://bjbms.org/ojs/index.php/bjbms/article/view/9489/2929

## Data Availability

The data used to support the findings of this study have come from The Cancer Genome Atlas (TCGA) database (http://cancergenome.nih.gov) (phs000178).
